# Micronodular Thymoma with Lymphoid Stroma: A Mediastinal Mass Masquerading as a Pericardial Mass

**DOI:** 10.14797/mdcvj.516

**Published:** 2021-10-19

**Authors:** Kent Swimley, Hilda M. Gonzalez-Bonilla, Alpana Senapati, Karla M. Kurrelmeyer

**Affiliations:** 1 Houston Methodist Hospital, Houston, Texas, US; 2 Howard University Hospital, Washington, DC; 3 Intermountain Heart Institute Cardiology-Intermountain Medical Center, Murray, Utah, US; 4 Houston Methodist DeBakey Cardiology Associates, Houston, Texas, US

**Keywords:** micronodular thymoma, lymphoid stroma, mediastinal mass, micronodular thymoma

## Abstract

A 61-year-old man presented to the emergency room with lower extremity edema. Physical exam was only remarkable for a diastolic murmur in the right carotid area and left lower extremity edema. Venous Doppler revealed a deep venous thrombosis in the left lower extremity. Chest computed tomography (CT) with intravenous contrast ruled out pulmonary embolism but showed a mediastinal mass adjacent to the pericardium. Further imaging with cardiac magnetic resonance imaging (CMR) and cardiac CT angiography (CCTA) enabled localization and evaluation of the structural characteristics of the mass. The decision was made to excise the mass due to increasing size compared with its measurements on prior chest CTs and a high degree of vascularization seen on CMR and CCTA, which was concerning for an enlarging arteriovenous malformation or a hemangioma. However, histopathologic analysis of the mass revealed it to be a micronodular thymoma.

## Background

Mediastinal, cardiac and pericardial masses are rare. These tumors can be primary or metastatic, and the differential diagnosis is broad. Primary cardiac tumors have a reported prevalence in autopsy series of 0.001% to 0.03%.^1^ Pericardial tumors are less common, making up only 6.7% to 12.8% of all primary cardiac tumors.^2^,^3^ Most of these tumors are found incidentally on imaging, and symptoms vary depending on the location of the tumor. Echocardiogram is the initial test for diagnosis of pericardial tumors; however, cardiac computed tomography angiography (CTA) and cardiac magnetic resonance imaging (CMR) offer higher spatial resolution and tissue characterization, thereby helping narrow the differential diagnosis and assist in operative planning. Patients with cardiac, pericardial, and mediastinal masses should undergo multimodality imaging to determine if they would benefit from surgical excision and, if so, delineate the best surgical approach.

## Presentation

A 61-year-old man with hypertension presented to the emergency room with a 3-day history of left lower extremity edema. The patient denied trauma, chest pain, dyspnea, or palpitations. His vital signs on initial evaluation were heart rate 85 bpm, blood pressure 169/92 mm Hg, respiratory rate 18 bpm, temperature 97.7 °F, and oxygen saturation 97% on room air. Physical exam was remarkable for left lower extremity edema and a diastolic bruit in the right carotid area.

## Investigations

**Figure 1:**
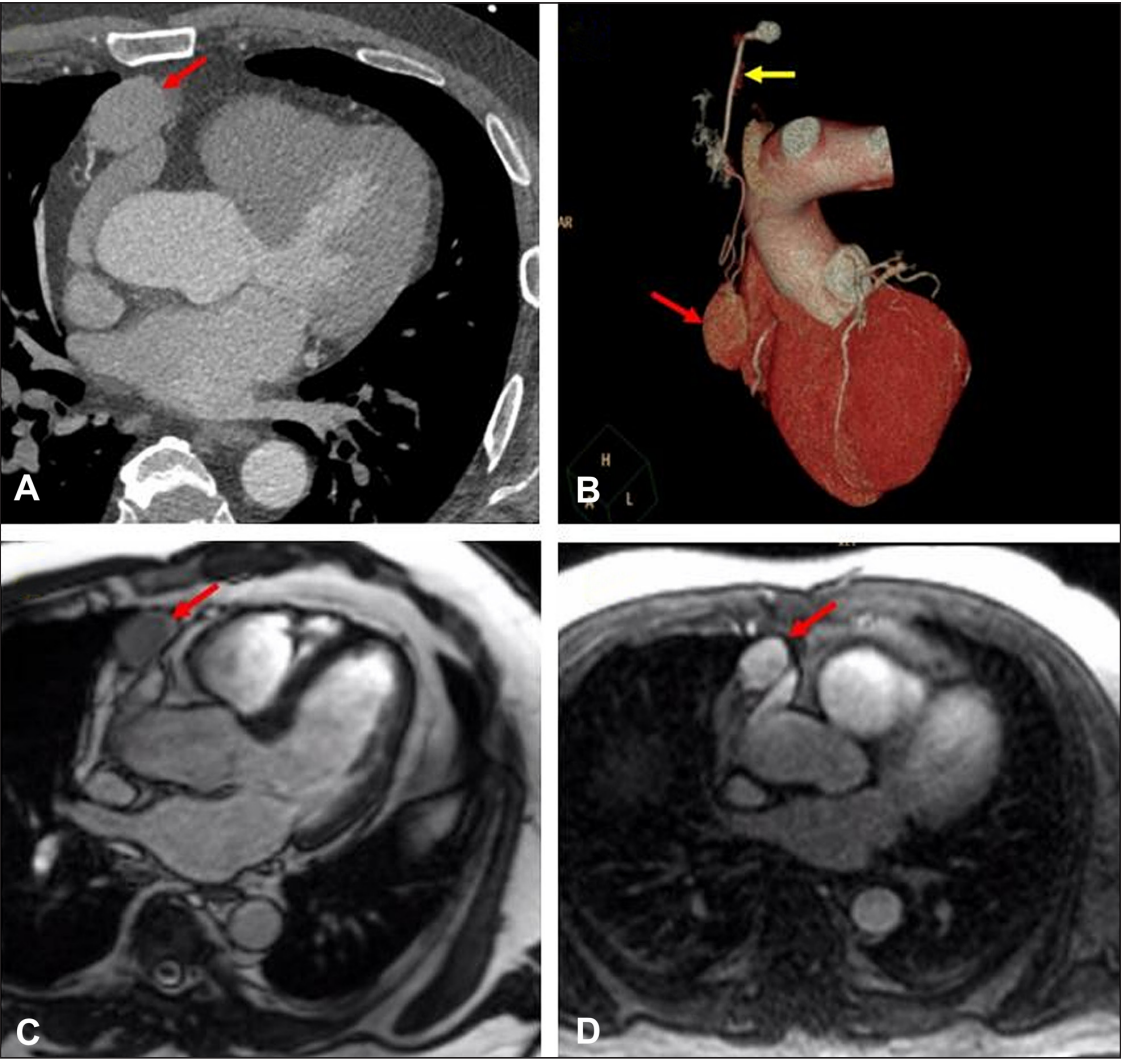
**(A)** Axial cross section on cardiac CTA showing the well circumscribed mass (red arrow) anterior to the right atrial appendage and adjacent to the pericardium. **(B)** A 3-dimensional reconstructed CT image of the mass with arterial supply from the right internal mammary artery (yellow arrow). **(C, D)** CMR images show the mass with significant first-pass perfusion and late gadolinium enhancement consistent with a highly vascularized structure.

Doppler ultrasound of the left lower extremity showed a deep venous thrombosis (DVT). He had a contrast chest CT that did not show pulmonary emboli but demonstrated a 3 × 2 cm anterior mediastinal mass adjacent to the pericardium (***Figure 1***). On further review of prior imaging, the mediastinal mass was found on chest CTs 1 and 8 years prior to this presentation, albeit at a much smaller size. For further characterization of the mass, the patient underwent CMR, which revealed a large, well-circumscribed mass measuring 3.3 × 2.1 cm anterior to the right atrial appendage adjacent to the pericardium. Tissue characteristics showed significant first-pass perfusion with late gadolinium enhancement uptake and hyperintensity on T2 imaging relative to the myocardium. These findings are consistent with a highly vascular structure. There was no evidence of tissue invasion (***Figure 1***). Due to concerns for an arteriovenous malformation (AVM), he underwent CTA with 0.5-mm sequential cuts, which demonstrated a large 4 × 2.5 cm AVM in the anterior mediastinum that was supplied by branches off the right internal mammary artery and the left internal thoracic vein arising from the left brachiocephalic vein (***Figure 1***).

## Differential Diagnosis

Multimodality imaging allowed significant narrowing of the differential diagnosis. Because the initial chest CT did not reveal a fluid component, pericardial and thymic cysts were excluded from the differential diagnosis. Likewise, calcification was not seen on CT, therefore excluding teratomas, the most common type of germ cell tumor in the anterior mediastinum. CMR tissue characterization revealed no evidence of fat, which excluded lipomas, liposarcomas, and a localized epicardial fat pad. Lymphoma also was excluded based on multimodality imaging, which revealed no lobulated contour, hemorrhage, or necrosis within the mass or an accompanying pericardial effusion. CMR did reveal that the mass was highly vascularized, suggesting a hemangioma. CCTA likewise identified the blood supply to the mass, suggesting that the mass could be a large AVM. Thymomas can have a varied appearance on multimodality imaging. They always appear as a well-circumscribed solid mass, but less than a third will have hemorrhage and necrosis, less than 20% will have peripheral calcification, and some will appear as highly vascularized masses.

## Treatment

**Figure 2:**
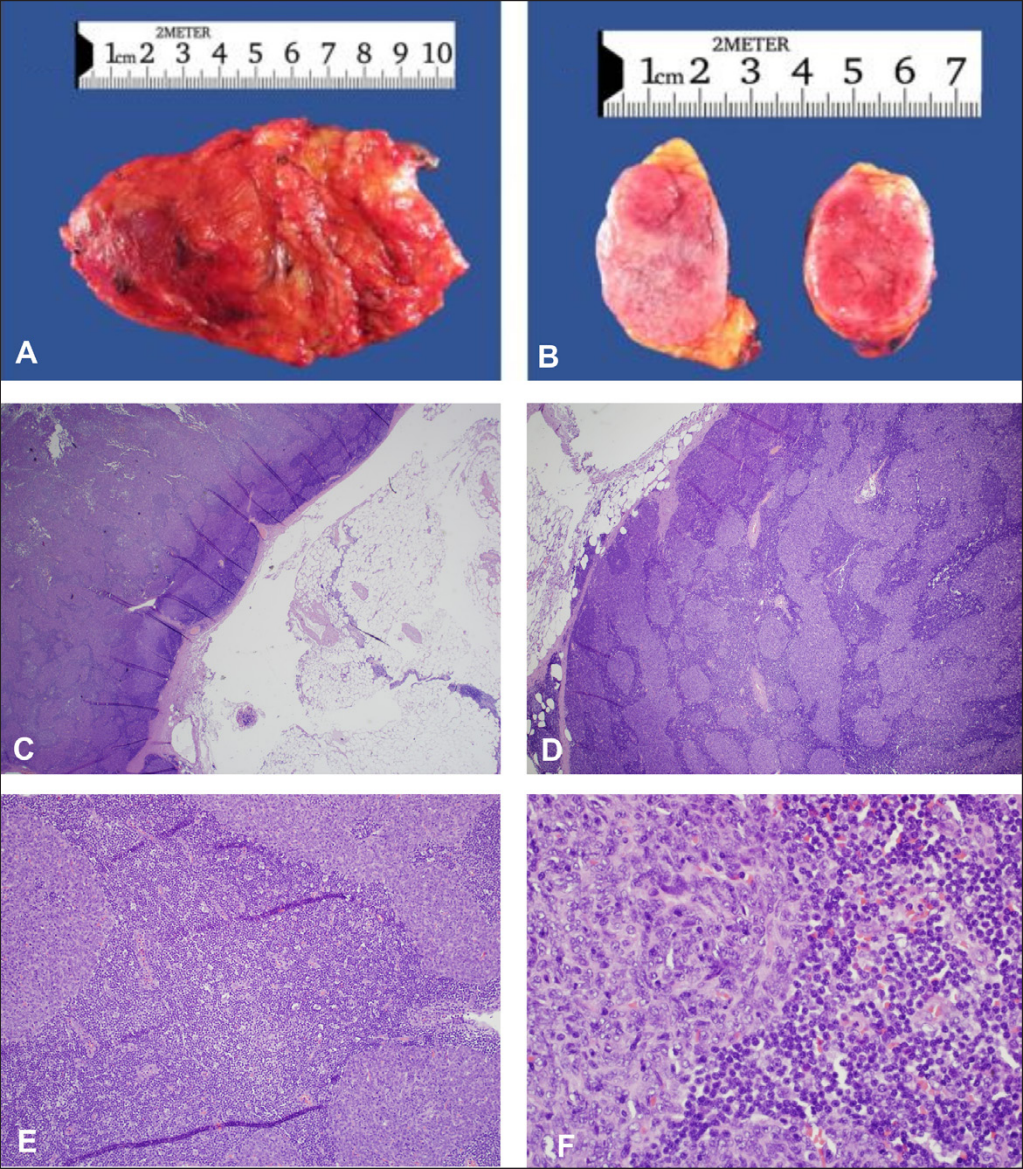
Morphologic analysis of the mediastinal mass. **(A)** On gross examination, the specimen consists of a single portion of fibroadipose tissue (10.5 × 7.5 × 1.8 cm; 65 gm). **(B)** Serial sectioning reveals a well-circumscribed, tan-red mass (4.0 × 2.4 × 2.0 cm) that is predominantly solid with some focal cystic areas. The margins appeared grossly free of tumor. **(C)** The tumor is composed of one large, solid nodule with a thin, fibrous capsule abutting fatty, fibrous thymic tissue. **(D)** The tumor nodule is characterized by multiple variably sized tumor nests surrounded by an abundant lymphoid stroma. **(E)** There is a distinct boundary between medullary-type epithelial cells and lymphoid stroma. **(F)** Tumor nests are composed of spindled to ovoid cells with moderate atypia and rare mitoses.

**Figure 3:**
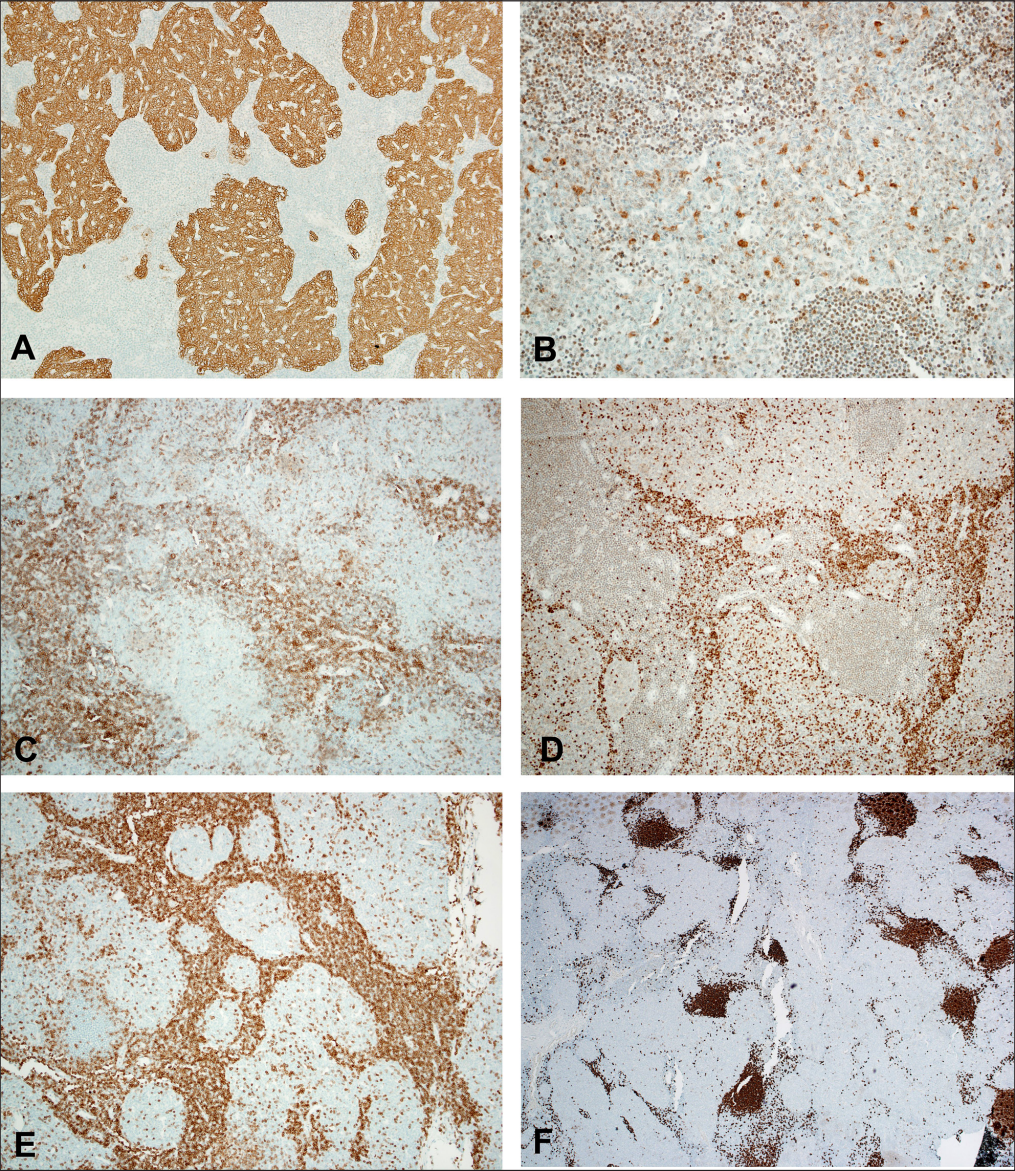
Immunostaining staining of the mediastinal mass. **(A)** The epithelial cells in the tumor nest are diffusely positive for CK (AE1/AE3), **(B)** patchy positive for CD117, and **(C)** negative for CD5. **(D)** Stromal T lymphocytes are positive for TDT and **(E)** CD3. **(F)** CD20 highlights mature B cells within the lymphoid stroma.

After the patient was evaluated for mass resection, he underwent surgery with localization and coil embolization of the feeding artery coming off the right internal mammary artery supplying the mass. Using cone beam CT and laser guidance, the mediastinal mass was excised using a minimally invasive procedure. The mass measured 4 × 2 cm and was sent for frozen section diagnosis followed by submission for formalin-fixation and paraffin embedding. Hematoxylin and eosin staining (H&E, ***Figure 2***) was followed by multiple immunohistochemical stains (IHC, ***Figure 3***) for more accurate characterization of the tumor. Histopathologic analysis diagnosed the mass as a micronodular thymoma (MNT).

Fortunately, after tumor excision, our patient has continued to do well without further symptoms and has received follow-up in the clinic every 3 months.

## Discussion

Grossly, MNT can have variable morphology ranging from solid to cystic architectural patterns. Microscopically, MNT has a characteristic histological pattern that is described as multiple small tumor islands surrounded by abundant epithelial cell-free lymphoid stroma consisting of mature B and T cells. However, multiple studies have shown that although the histological pattern remains the same, the morphology can be highly variable.^4^

In addition to morphology, review of the literature has shown the tumor cells of MNT have variable immunophenotypic expression. For example, CD5 and CD117 staining has been shown to vary from negative to focally and diffusely positive.^5^ Some of these tumors can have a vascular phenotype. However, no study has considered this characteristic as part of the stratification or classification. The A and AB thymomas can be characterized by a dense network of capillary-like vessels with pericyte coverage, whereas B thymomas can show a loose vascular network with increased vascular diameters. This was not the case in our patient even though the mass appeared highly vascular on imaging and initially was diagnosed as a hemangioma or an AVM.

The World Health Organization classification is currently used to morphologically classify thymomas; however, other histological classification systems have been proposed, including the Modified Masaoka Staging system and proposed Suster and Moran classification system.^4^ Due to problematic interobserver reproducibility in determining encapsulated versus minimally invasive thymomas, the current (8th) edition of the American Joint Committee of Cancer and Union (AJCC) does not distinguish between encapsulated and minimally invasive thymomas.^6^ However, additional studies are needed to determine whether the AJCC staging system is prognostically superior to the modified Masaoka staging system.

Our case had microscopic capsular invasion, placing it in the stage IIA category for modified Masaoka staging but only stage I in the TNM AJCC staging system. For MNT specifically, a review of the literature by Qu et al. shows that, regardless of MNT pathology or stage, this unique thymoma subtype has a uniformly favorable prognosis. In general, surgery remains the standard of care for encapsulated thymomas and has high cure rates.

There are no specific imaging guidelines for evaluating pericardial masses. The American Society of Echocardiography states that echocardiography remains the initial imaging test for evaluation of pericardial masses because of its ease of use, availability, and cost effectiveness. However, multimodality imaging plays an integral role in evaluating cardiac masses because of its superior spatial resolution and tissue characterization capabilities.^7^ CCTA and CMR help determine the presence of fluid, fat, calcification, and thrombus within the mass. They also help identify infiltration of adjacent structures and determine the mass’s vascular supply, which are both important in surgical planning.^8^,^9^ Multimodality imaging helps narrow the differential diagnosis, but the final diagnosis ultimately depends on detailed and extensive histopathological analysis.
